# Whole-Genome Sequence of *Burkholderia* sp. Strain RPE67, a Bacterial Gut Symbiont of the Bean Bug *Riptortus pedestris*

**DOI:** 10.1128/genomeA.00556-14

**Published:** 2014-06-19

**Authors:** Kazutaka Takeshita, Tomoko F. Shibata, Naruo Nikoh, Tomoaki Nishiyama, Mitsuyasu Hasebe, Takema Fukatsu, Shuji Shigenobu, Yoshitomo Kikuchi

**Affiliations:** a Bioproduction Research Institute, Hokkaido Center, National Institute of Advanced Industrial Science and Technology, Sapporo, Japan; b National Institute for Basic Biology, Okazaki, Japan; c Department of Liberal Arts, the Open University of Japan, Chiba, Japan; d Advanced Science Research Center, Kanazawa University, Kanazawa, Japan; e School of Life Science, Graduate University for Advanced Studies, Okazaki, Japan; f Bioproduction Research Institute, National Institute of Advanced Industrial Science and Technology, Tsukuba, Japan

## Abstract

*Burkholderia* sp. strain RPE67 is a bacterial symbiont isolated from a field-collected bean bug, *Riptortus pedestris*. To understand the genetic basis of the insect-microbe symbiosis, we performed whole-genome sequencing of the *Burkholderia* strain, revealing an 8.69-Mb genome consisting of three chromosomes and three plasmids.

## GENOME ANNOUNCEMENT

The bean bug *Riptortus pedestris*, known as a serious pest of leguminous crops, develops sac-like tissues called crypts at the posterior region of the midgut, the lumen of which is densely colonized by a *Burkholderia* symbiont belonging to the *Betaproteobacteria* (1). The symbiont is acquired every generation from the ambient soil (2) and enhances host growth and fecundity (2, 3). Here, we announce the complete genome sequence of *Burkholderia* sp. strain RPE67, isolated from the symbiotic organ of a field-collected *R. pedestris* bug (4). This is the second whole-genome sequence of a *Burkholderia* symbiont, following that of *Burkholderia* sp. strain RPE64 (5).

Whole-genome sequencing of RPE67 was performed with a PacBio RS (Pacific Biosciences). Approximately 10- and 20-kb insert libraries were constructed with a DNA template prep kit 2.0 and sequenced using C2 chemistry with XL and P4 polymerase (after version up to RS II) using 2 and 4 SMRT cells, yielding 100,396 and 298,641 reads with 2.1- and 4.8-kb mean maximum subread lengths (https://github.com/tfshibata/Burkholderia_rpe67_stats), respectively. In total, 1.87 Gb of independent fragment reads were collected (GenBank accession no. DRA002232), corrected for sequencing errors with sprai version 0.9.5 (http://zombie.cb.k.u-tokyo.ac.jp/sprai/), and assembled with Celera Assembler version 7.0 (6), yielding six contigs, of which five are circular. The contigs were polished with Quiver packaged in SMRT Analysis version 2.0.1 (7). Closing of the linear contig was accomplished by Sanger sequencing of the PCR products with an ABI 3130. Coding genes were predicted with Prodigal version 2.60 (8), and BLASTp (9) searches were performed against the UniProt TrEMBL database (release 2013_12) (10) for annotation. tRNAs and rRNAs were predicted with tRNAscan-SE version 1.3.1 (11) and RNAmmer version 1.2 (12), respectively.

The complete genome of *Burkholderia* sp. strain RPE67 is 8.69 Mb and consists of three circular chromosomes and three plasmids: chromosome 1 (3,090,091 bp, 2,859 protein-coding sequences [CDSs], 56 tRNAs, and 12 rRNAs), chromosome 2 (1,787,110 bp, 1,688 CDSs, 4 tRNAs, and 3 rRNAs), chromosome 3 (1,680,600 bp, 1,553 CDSs, 3 tRNAs, and 3 rRNAs), plasmid 1 (1,438,033 bp and 1,342 CDSs), plasmid 2 (495,745 bp, 476 CDSs, and 1 tRNA), and plasmid 3 (194,177 bp and 194 CDSs). The G+C content is 59.3% to 64.1%.

While RPE64 and RPE67 are associated with the same host species, the total genome size of RPE67 is 1.73 Mb larger than that of RPE64 (6.96 Mb, 3 chromosomes and 2 plasmids, and 6,732 CDSs) (5). While chromosomes 1 are similar in size and gene composition between the two strains, other chromosomes and plasmids differ substantially in size and gene compositions. Chromosomes 2 and 3 are 0.32 Mb and 0.78 Mb larger in RPE67, respectively. The OrthoMCL analysis (13) showed 5,151 orthologous groups shared by the symbiont strains, which include 5,438 CDSs in RPE67 and 5,323 CDSs in RPE64.

Members of the genus *Burkholderia* are remarkably diverse, and the whole genomes of 37 strains of human pathogenic, plant-associated, and environmental species have been sequenced (ftp://ftp.ncbi.nlm.nih.gov/genomes/Bacteria/). Genome comparisons between the insect-associated symbiont *Burkholderia* strains RPE67 and RPE64 and other strains should highlight key genes for insect-microbe mutualism and give us insights into the evolutionary origin of symbiotic association.

### Nucleotide sequence accession numbers.

The complete genome sequence of *Burkholderia* sp. RPE67 (including three chromosomes and three plasmids) has been deposited in DDBJ/EMBL/GenBank under accession no. AP014576 to AP014581.
